# Better grip force control by attending to the controlled object: Evidence for direct force estimation from visual motion

**DOI:** 10.1038/s41598-019-49359-8

**Published:** 2019-09-11

**Authors:** Shinya Takamuku, Hiroaki Gomi

**Affiliations:** NTT Communication Science Laboratories, 3‐1 Morinosato Wakamiya, Atsugi, Kanagawa 243-0198 Japan

**Keywords:** Computational neuroscience, Sensorimotor processing

## Abstract

Estimating forces acting between our hand and objects is essential for dexterous motor control. An earlier study suggested that vision contributes to the estimation by demonstrating changes in grip force pattern caused by delayed visual feedback. However, two possible vision-based force estimation processes, one based on hand position and another based on object motion, were both able to explain the effect. Here, to test each process, we examined how visual feedback of hand and object each contribute to grip force control during moving an object (mass) connected to the grip by a damped-spring. Although force applied to the hand could be estimated from its displacement, we did not find any improvements by the hand feedback. In contrast, we found that visual feedback of object motion significantly improved the synchrony between grip and load forces. Furthermore, when both feedback sources were provided, the improvement was observed only when participants were instructed to direct their attention to the object. Our results suggest that visual feedback of object motion contributes to estimation of dynamic forces involved in our actions by means of inverse dynamics computation, i.e., the estimation of force from motion, and that visual attention directed towards the object facilitates this effect.

## Introduction

Dexterous motor control often requires accurate estimation of interaction forces acting between our hand and the objects we control. When carrying a plastic cup filled with beer, for instance, you want to minimize your grip force to avoid spilling it. However, if the grip force is too weak, such that friction falls below the interaction force tangential to the gripped surface, the cup will slip out of your hand. Accordingly, the task requires accurate estimation of such interaction force referred to as the load force.

Studies on human grip force control have revealed our ability to accurately predict such load forces. When moving a rigid object, for instance, the grip force applied to the object is controlled to change in parallel with the acceleration-dependent load force^1,2^. Namely, grip force is timed to coincide with the increase and decrease of the load force, and peak grip force correlates with the peak load force. Furthermore, such accurate adjustment of grip force generalizes to cases with various dynamics in which the dynamic load force of the object depends on position and/or velocity^3^.

How is such accurate load force prediction achieved? Obviously, the prediction needs to be constantly adjusted based on sensory feedback. Previous studies^1,4^ highlighted contribution of somatosensory feedback for this purpose. For instance, a study^1^ showed that cutaneous anaesthesia of index finger and thumb impairs adaptation of grip force to frictional condition. Proprioceptive contribution was also suggested by examining grip force changes caused by perturbations applied to the arm^4^.

**Figure 1:**
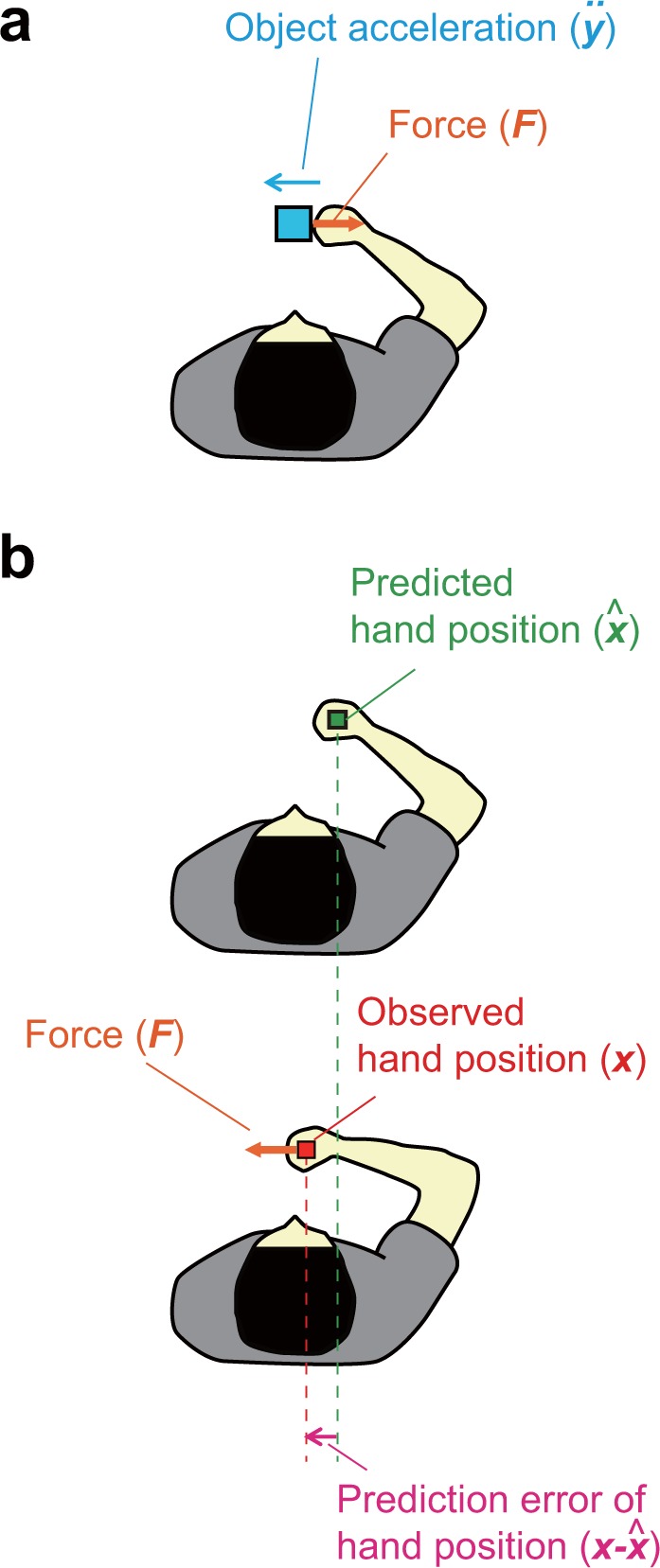
Potential contribution of visual feedback. (**a**) Potential contribution of object feedback. When moving a rigid object (mass), its reaction force linked to its inertia (*F*) can be estimated from its acceleration ($\ddot{y}$). (**b**) Potential contribution of hand feedback. When force (*F*) is applied to the hand, observed position of the hand (*x*) would be shifted from its natural position ($\hat{x}$) that can be predicted based on internal models of the body. The applied force can be estimated from the displacement (${\rm{x}}-\hat{x}$) by considering the stiffness characteristics at the hand position.

Interestingly, recent studies have suggested contribution of visual feedback to the force estimation. Sarlegna *et al*.^5^ showed that delayed visual feedback of grip position influences grip force control. Here, participants gripped an end of a spring of which the other end was fixed (see section S1.2 in the Supplementary Information). When they repeatedly stretched the spring, the grip force changed in correlation with the elastic load force with slight temporal precedence (phase difference between the two forces). However, when the motion of the cursor which indicated the grip position was delayed, the precedence of the grip force increased in correlation with the amount of the introduced delay. The authors assumed that the participants interpreted the cursor as an object (mass) attached to the grip with a damped spring and changed their grip force to cope with the presumed increase in load force corresponding to its inertia. The hypothesis that humans estimate inertia of a delayed cursor was recently supported by our study^6^. In this study, we showed that a peculiar resistive sensation, experienced while moving a delayed cursor, correlates with the amount of exposure to the forward acceleration of the cursor. Since forward acceleration of a carried rigid object correlates with its inertial reaction force (remember the Newton’s laws of motion), the evidence suggests that the sensation corresponds to the inertial force of the cursor. Taken together, these observations would imply that visual feedback of *object motion* contributes to the force estimation by means of inverse dynamics computation, i.e., estimation of dynamic forces from observed object motions, as illustrated in Fig. 1a.

Another line of evidence, however, suggested a different story. Models of force-field adaptation generally assumed that we estimate the strength of the perturbation force from the prediction error of hand position it causes^7,8^. Similarly, studies in the field of human-computer interface have anecdotally reported that perturbation of cursor position causes an illusory sensation of force applied to its direction^9,10^. In line with these views, Honda *et al*.^11^ found that delaying visual motion of a hand carrying an object causes the object to be perceived as heavier than the case without the delay. Based on further finding that this bias is decreased after adapting to the visual delay, they argued that prediction error of hand position causes the effect. When external force is applied to the hand, observed position of the hand would be shifted from its natural position that can be predicted from internal models of body dynamics as illustrated in Fig. 1b. If one has an internal representation of the stiffness ellipse at the hand position, the applied force can be estimated from the displacement of the hand. Therefore, visual feedback of *hand position* can also contribute to the force estimation. Importantly, temporal precedence of grip force relative to the load force and its correlation with the introduced delay, described earlier, can also be explained by a hypothesis that participants assumed an additional load force proportional to the error of cursor position caused by the delay (see S1 in the Supplementary Information).

The difficulty of understanding the visual contribution is linked to the excessive use of delayed visual feedback in the existing studies. Although, many studies have explored how our brain internally represents the delay, no study to date has succeeded in providing a consistent picture. While a group of studies^5,6,12^ have suggested that the delayed cursor is interpreted as an *object* connected to the hand with a certain mechanical system, others have favored the view that the cursor is interpreted as indicating the *hand* position^11,13^.

Here, to directly test the two possible visual contributions to the force estimation, we examined a case where the hand and the object are not rigidly coupled and each can be displayed separately. Namely, participants moved a rigid object attached to the grip with a damped spring (i.e., a simulated spring-mass damper system). The possible contributions of visual feedback of hand and object (cursors indicating each position) to the estimation of the load force (inertial reaction force of the object) were then tested based on two experiments under this situation. In the first experiment, we examined how showing either or both the hand cursor and the object cursor improved the grip-load force coupling (i.e., adjustment of the grip force pattern to the load force pattern). Note that we used normal feedback instead of the delayed feedback that was used in the earlier studies. Showing the object cursor improved the temporal alignment of grip and load forces, whereas displaying the hand cursor did not. Since showing both cursors also did not improve the synchrony, we run a second experiment to test the possible role of visual attention. Improvement in temporal alignment of the grip and load forces were observed only when the participants were instructed to direct their attention to the object. Our finding supports the hypothesis that dynamic forces involved in our action are directly estimated from visual motion of the controlled object by means of inverse dynamics computation and further suggests that the process is facilitated when visual attention is directed to the object.

## Results

**Figure 2:**
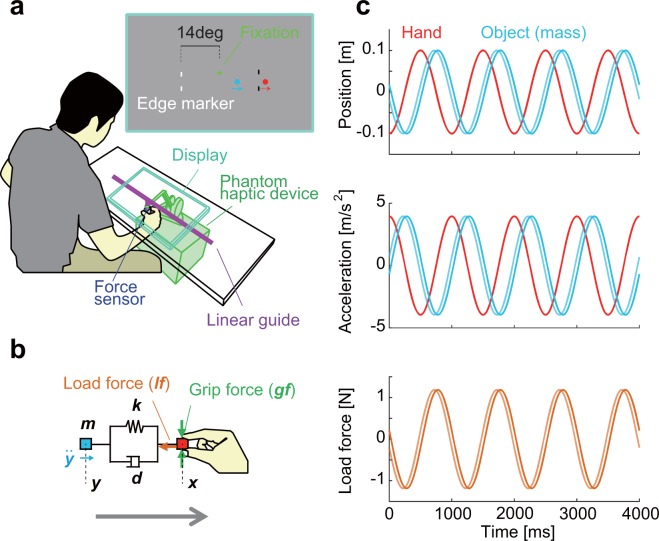
Setup, simulated system, and intended stimuli used in the experiment. (**a**) Illustration of setup used in the experiment. Right top panel illustrates what was displayed on the screen. (**b**) Simulated system. A rigid object (cyan) with mass of *m* [kg] was attached to the pinched cube (red) with a damped spring (stiffness: *k* [N/m], damping factor: *d* [Ns/m]). Load force correlated with the acceleration of the load ($\ddot{y}$). (**c**) Illustration of intended hand/object trajectories and load force patterns. Upper panel illustrates intended hand (red) and load trajectories (cyan) of each load force condition (light color: small delay condition, dark color: large delay condition). Middle and bottom panels illustrates the intended load accelerations and load forces respectively.

Figure 2 illustrates the design of our experiment. Participants pinched a cube attached to an end of a haptic device placed below a table (Fig. 2a). Two 3DOF force sensors were attached to the cube to measure the load and grip forces. They repeatedly moved the cube left-and-right-and-back with specified amplitude and rhythm (1 Hz specified by periodic beeps). Motion of a rigid object attached to the cube with a damped spring was numerically simulated, and its inertial reaction force was applied to the cube (Fig. 2b). Mechanical property of the spring was set such that the object would follow the hand with a phase delay of either 80 or 100 degrees (small and large delay conditions illustrated as light and dark colored trajectories in Fig. 2c). Since the delay was chosen randomly for each trial, participants had to adjust the temporal pattern of their grip force to match that of the load force. Temporal alignment of the grip and load forces was measured by calculating the temporal discrepancy between the two forces (i.e., temporal displacement which maximizes the cross-correlation between the two forces). Since the changes in grip force preceded the changes in load force in all the conditions observed, the temporal discrepancy would be referred to as the temporal precedence of grip force.

Visual feedback of hand and object position was displayed as red and blue filled circles (cursor) on a LCD monitor placed horizontally above the table (Fig. 2a). To control for eye movements and visual input, participants were instructed to fixate on a fixation point shown at the center of the screen in both experiments. Two markers (edge markers) were shown at the left and right side of the screen to indicate the specified amplitude of the hand trajectory.

### Experiment 1: Effects of hand and object visual feedback on the grip force coordination

In the first experiment, we examined how visual feedback of hand and object positions contribute to the adjustment of the grip force. We examined four visual feedback conditions: condition with only the hand cursor (hand condition), condition with only the object cursor (object condition), condition with both hand and object cursors (both condition), and condition without any cursors (control condition). In all these conditions, participants were instructed to move the cube with specified amplitude and movement cycle. In the object condition, they were asked to simultaneously control the object so that it would also move with the same amplitude and movement cycle. Combined with the load force condition, there were eight conditions in total. The order of the conditions was pseudo-randomized across trials.

**Figure 3:**
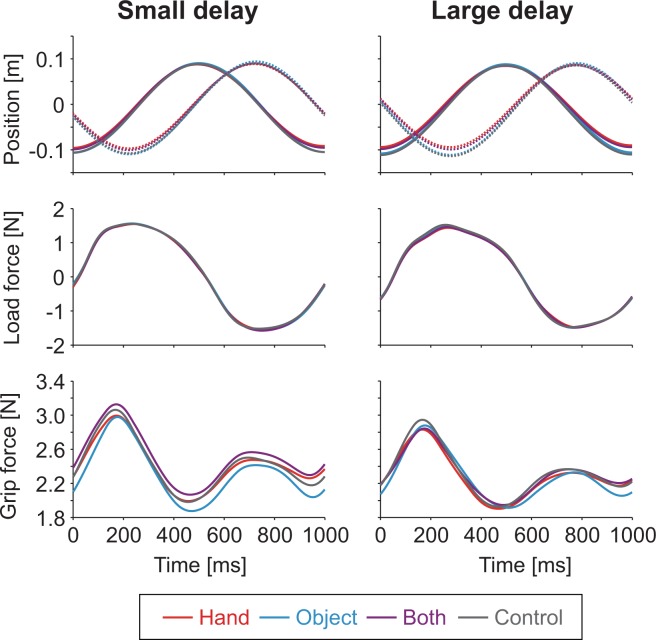
Hand trajectory (top panel), load force pattern (middle panel), and grip force pattern (bottom panel) of a typical participant averaged across all cycles of each visual feedback condition examined in experiment 1. Colors indicate visual conditions (red: hand, cyan: object, purple: both, gray: control). Left and right panels indicate data for small and large delay conditions respectively.

Figure 3 shows recorded hand and object trajectory (top panel), load force pattern (middle panel), and grip force pattern (bottom panel) of a typical participant averaged across all cycles of each condition. Data for the small and large delay conditions are shown in the left and right panels respectively, and the colors indicate the visual conditions (red: hand, cyan: object, purple: both, gray: control). In all the observed conditions, the object followed the hand approximately as intended. Amplitude ratio between object and hand trajectories, averaged across all conditions, was 1.0 ± 0.01 and delay of the object position relative to the hand position, averaged across all visual conditions, was 218 ± 0.5 and 271 ± 0.8 ms (corresponding to phase delays of 78.3 ± 0.8 and 97.7 ± 1.2 degrees) for the small and large delay conditions, respectively (mean ± s.e. across participants).

**Figure 4:**
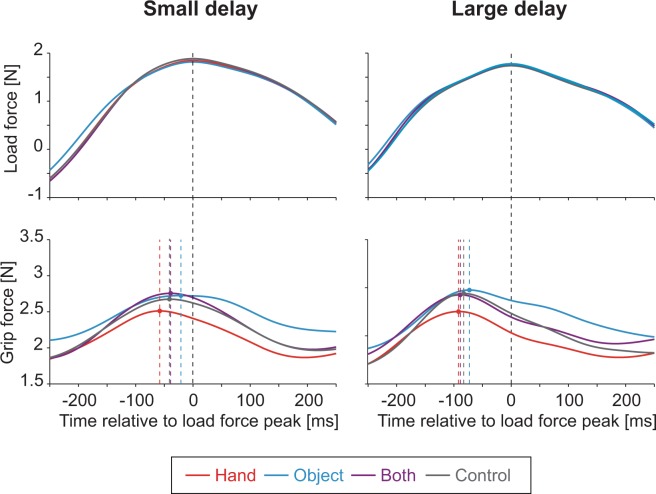
Load force pattern (top panel) and grip force pattern (bottom panel) of a typical participant aligned with time of load force peak and averaged across all peaks of each visual feedback condition examined in experiment 1 (red: hand, cyan: object, purple: both, gray: control). Left and right panels indicate data for small and large delay conditions respectively. Dotted lines indicate times when average grip force peaked in each condition.

Load force peaked approximately when the object was at the left and right edges (corresponding to the positive and negative peaks). Although absolute load force peaked twice in each cycle, peak of grip force was more evident when the object was at the left edge. Figure 4 shows load force pattern (top panel) and grip force pattern (bottom panel) of a typical participant aligned with the time when the load force peaked and averaged across all peaks of each visual condition examined in experiment 1. As mentioned above, changes in grip force tended to precede the changes in load force in all the conditions observed in our study. Importantly, grip force not only increased before the load force started increasing, but it also decreased while the load force was still increasing. The grip forces measured when the load force peaked was significantly smaller than its peak values (the ratios averaged across trials and participants ranged from 87.6% to 90.0% in experiment 1 and from 88.2% to 92.0% in experiment 2). This led the ratio of grip force relative to the load force to be smaller than the case where the two forces are synced and increased the risk that the object would slip. Accordingly, the temporal precedence of the grip force suggested suboptimal performance in grip force control.

Cross-correlation analysis was applied to the load and grip forces to estimate the accuracy of the load force prediction within each condition. Two-factor repeated measures ANOVA revealed no significant effect of visual condition on the maximum correlation between the two forces (*F*_3,33_ = 0.26, *p* = 0.85, ${\eta }_{p}^{2}=0.02$). Meanwhile, we found significant differences in the temporal relationships between the two forces.

**Figure 5:**
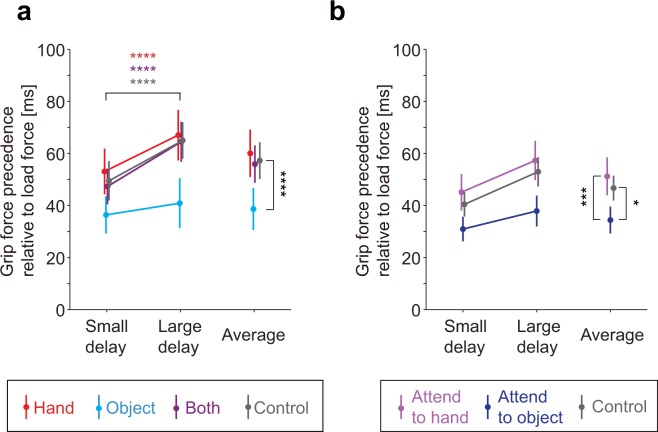
Temporal precedence of grip force relative to load force averaged across all participants in experiment 1 (**a**) and 2 (**b**). Colors indicate visual condition. Error bars denote standard errors across participants. Asterisks on the horizontal bar indicate significant effect of load force conditions within the visual condition indicated by the color. Asterisks along the vertical bars indicate significant difference between the visual conditions. Multiple comparison was controlled based on the Ryan’s method^14^. *, **, ***, and **** denote *p* < 0.05, *p* < 0.01, *p* < 0.005, and *p* < 0.001, respectively.

Figure 5a shows the temporal precedence of grip force relative to the load force for the conditions in experiment 1 associated with maximal cross-correlation between the two forces (mean ± s.e. across participants). The precedence of the grip force in the control condition was 49.5 ± 7.5 ms for the small delay condition and 65 ± 7.0 ms for the large delay condition. These precedencies were both significantly larger than 0 (small delay: *t*_11_ = 6.56, *p* = 4.09 × 10^−5^, *d* = 1.89; large delay: *t*_11_ = 9.31, *p* = 1.51 × 10^−6^, *d* = 2.69), suggesting that the timing of the grip force was suboptimal in these cases. The difference of the precedence between the two load force conditions (15.5 ± 3.7 ms) was significantly smaller (*t*_11_ = 5.33, *p* = 2.42 × 10^−4^, *d* = 1.54) than the difference of the delay of the measured load force (33.8 ± 9.8 ms). This suggests that participants were able to compensate for the change in load force timing to some extent without visual feedback. However, the difference of the precedence between the two load force conditions were significantly larger than 0 (*t*_11_ = 4.19, *p* = 1.50 × 10^−3^, *d* = 1.3), suggesting that the compensation was incomplete.

Two-factor repeated measures ANOVA applied to the precedence revealed main effects of visual (*F*_3,33_ = 5.28, *p* = 4.36 × 10^−3^, ${\eta }_{p}^{2}=0.32$) and load force (*F*_1,11_ = 22.1, *p* = 6.49 × 10^−4^, ${\eta }_{p}^{2}=0.67$) conditions as well as the interaction between the two conditions (*F*_3,33_ = 3.51, *p* = 2.58 × 10^−2^, ${\eta }_{p}^{2}=0.24$). Post-hoc analysis (Ryan’s method^14^) among the visual conditions showed that the precedence was smaller in the object condition (*t*_11_ = 3.80, *p* = 3.16 × 10^−4^, *d* = 1.10) compared to the control condition, but not in the hand (*t*_11_ = 0.33, *p* = 0.75, *d* = 0.10) or in the both (*t*_11_ = 0.08, *p* = 0.93, *d* = 0.02) condition.

The precedence was significantly larger in the large delay condition compared to the small delay condition not only in the control condition, but also in the hand (*t*_11_ = 4.69, *p* = 6.62 × 10^−4^, *d* = 1.35) and both (*t*_11_ = 7.10, *p* = 2.0 × 10^−5^, *d* = 2.05) conditions. In contrast, the difference was not significant in the object condition (*t*_11_ = 0.84, *p* = 0.42, *d* = 0.24). Furthermore, the difference of the precedence was smaller in the object condition when compared with the control condition (*t*_11_ = 2.33, *p* = 3.96 × 10^−2^, *d* = 0.67), but not in the hand (*t*_11_ = 0.35, *p* = 0.73, *d* = 0.1) and both (*t*_11_ = 0.51, *p* = 0.62, *d* = 0.15) conditions. These results suggest that the grip-load force coordination improved only in the object condition.

While visual condition changed the grip-load force coordination, it could have also changed the hand trajectory. In order to rule out the possibility that the differences in the hand trajectory caused the difference in grip-load force coordination, we analyzed its kinematic aspects. Amplitude of hand trajectory, averaged across the load force conditions, tended to be significantly (p < 0.0005) wider in the object (107 ± 2.3 mm) and control (105 ± 2.4 mm) conditions compared to the hand (101 ± 2.3 mm) and both (100 ± 2.0 mm) conditions (mean ± s.e. across participants; example shown in Fig. 3), possibly due to the lack of hand visual feedback in these conditions. Meanwhile, the difference between the object and control conditions was not significant (*t*_11_ = 1.13, *p* = 0.28, *d* = 0.33). Movement cycle of the hand, averaged across the load force conditions, was significantly (p < 0.01) larger in the object condition (999 ± 3 ms) compared to the other conditions (hand: 985 ± 6, both: 989 ± 5, control: 991 ± 5 ms). This increase in movement cycle, however, did not correlate with the decrease in grip precedence (correlation analysis based on difference between object and control conditions: R = −8.6 × 10^−3^, *p* = 0.98). Timing of the hand trajectory relative to the beeps (definition given in the Data analysis section) did not depend on the visual condition.

### Experiment 2: Effect of visual attention on the grip force coordination

While our first experiment showed that providing only the object cursor improves the temporal alignment of grip and load forces, the improvement was not observed when both the hand and object cursors were visible. A possible account for this was that visual attention needs to be directed to the object cursor to cause a detectable improvement, but that was not the case in the both condition since the participants were instructed to move the hand with specified rhythm and width. In order to test the possible role of visual attention, we run the second experiment.

Here, participants moved the cube left-and-right-and-back either with both the hand and object cursors (both condition) or without them (control condition). In the both condition, they were instructed to direct their attention either to the hand cursor (attend-to-hand condition) or to the object cursor (attend-to-object condition). In summary, there were three visual conditions and the conditions were specified by the color of the fixation point (red: attend-to-hand, blue: attend-to-object, black: control). Combined with the two load force conditions, there were six conditions in total.

Again, maximum correlation between the load and grip forces did not depend on the visual condition (*F*_2,30_ = 0.03, *p* = 0.97, ${\eta }_{p}^{2}=0.002$), but the temporal relationship depended on the condition. Figure 5b shows the grip force precedence relative to the load force associated with maximal cross-correlation between the two forces (mean and s.e. across participants). Two-factor repeated measures ANOVA revealed main effects of visual (*F*_2,30_ = 6.89, *p* = 3.45 × 10^−3^, ${\eta }_{p}^{2}=0.31$) and load force (*F*_1,15_ = 20.8, *p* = 3.73 × 10^−4^, ${\eta }_{p}^{2}=0.58$) conditions. Post-hoc analysis (Ryan’s method) among the visual conditions showed that the temporal precedence was smaller than the control condition when attention was directed towards the object feedback (*t*_15_ = 2.62, *p* = 1.35 × 10^−2^, *d* = 0.65), but not when it was directed towards the hand feedback (*t*_15_ = 0.96, *p* = 0.34, *d* = 0.24). The precedence differed significantly by visual attention (difference between both conditions: *t*_15_ = 3.12, *p* = 7.0 × 10^−4^, *d* = 0.78). These results support our hypothesis that visual attention directed to the object facilitates the improvement in grip force timing.

Amplitude of the hand trajectory was smaller in the two conditions with visual feedback compared to the control condition where the hand was invisible (both attending to hand: *t*_15_ = 4.27, *p* = 1.77 × 10^−4^, *d* = 1.07, both attending to object: *t*_15_ = 3.92, *p* = 4.43 × 10^−4^, *d* = 0.98). Meanwhile, there were no significant difference in the amplitude by attention (*t*_15_ = 0.33, *p* = 0.74, *d* = 0.08). Neither the load force condition nor the visual condition had a significant effect on the movement cycle of the hand.

## Discussion

Earlier studies have demonstrated that visual feedback influences our force estimation. There were two hypotheses on how the feedback could contribute to the estimation. One line of evidence suggested essentially that visual feedback of object motion contributes to the estimation by providing information of dynamic force based on inverse dynamics computation. Another line of evidence suggested that visual feedback of hand position contribute to the estimation by providing information on forces applied to the hand based on prediction errors of the hand position. The prediction of the hand position is based on forward computation. Here, we tested the two hypotheses by examining whether viewing (experiment 1) or directing attention to (experiment 2) either hand or object feedback would improve the grip-load force coupling when moving a rigid object attached to the grip with a damped spring.

Our first experiment showed that the synchrony between grip and load forces improved when object feedback was provided (Fig. 5a: difference between object and control conditions). Meanwhile, our experiment did not reveal any improvement in grip-load force coupling when the hand feedback was provided (no improvement in maximum cross-correlation or the temporal alignment between grip and load forces under the hand and both conditions). In the second experiment, we examined how directing attention to either the hand or the object cursor while showing both cursors influences the grip-load force coupling. The improvement in the temporal alignment of grip and load forces was observed only when the participants were instructed to direct their attention to the object cursor (Fig. 5b: difference between the attend-to-object and the control conditions). These results supported the hypothesis that visual feedback of object contributes to the force estimation.

### Grip force coordination when moving an object with a damped spring

As a basis of our observation, we found that coupling between grip and load forces were weak without visual feedback in the situation in which we tested our hypotheses. Cross-correlation analysis showed that the average temporal precedence of grip force relative to the load force was greater than 40 ms in all conditions without visual feedback (mean ± s.e. across participants in the control condition of experiment 2: 40.5 ± 4.7 ms for the small delay condition and 53.0 ± 5.6 ms for the large delay condition). This was significantly larger compared to the temporal discrepancy measured when participants directly pinched a rigid object of a similar mass and moved it side-by-side^2^. Similarly, maximal cross-correlation between the two forces were relatively low in our case (control condition in experiment1: R = 0.40 ± 0.04 for the small delay condition and R = 0.36 ± 0.04 for the large delay condition; control condition in experiment 2: R = 0.41 ± 0.04 for the small delay condition and R = 0.40 ± 0.04 for the large delay condition) compared to the case when the rigid object was held directly (R = 0.71).

It is still unclear why the grip-load force coordination was suboptimal in the observed situation. One factor which could explain the weak coupling is synergy between hand and arm muscles. An earlier study revealed a component of grip force which scales with the force produced by the arm muscles^15^. The component may explain the temporal discrepancy between the grip and load forces since the dynamic force of the object was relatively small and was not synchronized to the force patterns of the arm muscles. It may also explain the asymmetry in grip force pattern since different muscles are involved in the leftward and rightward movements. In line with this view, an earlier study have linked the asymmetry in grip force pattern to the synergy^16^. Beside this possibility, there is also a Bayesian account for explaining the grip precedence which we will discuss later. In any case, the observed situation involved a large margin for improving the grip-load force coupling, and thus provided a unique opportunity to explore the contribution of visual feedback in the load force estimation.

### Contribution of hand and object feedback in load force estimation

As described in the introduction, visual feedback of hand position can provide information on applied force by means of errors in its position. If the information is used for estimating the load force, this could improve the coupling between the grip and load forces. However, neither increase in maximal cross-correlation between the two forces nor improvement in synchrony between the two forces (Fig. 5a: difference between hand and control conditions) were observed in our experiments. While we do not have a conclusive account to why such contribution was not found in our study, there is a possibility that prolonged exposure to the load force may have updated the internal model of body dynamics and made it useless for estimating the applied forces. Note that the assumed framework for force estimation works only when the internal model predicts hand position without any load force.

In contrast, we found evidence for contributions of object feedback in the force estimation. In our first experiment, we found that visual feedback of object motion decreased the temporal discrepancy (precedence of grip force) between the grip and load forces (Fig. 5a: difference between object and control conditions). Furthermore, while delaying the load force timing increased the grip precedence in most conditions, such increase was not detected and significantly smaller in the object condition. This suggests that visual feedback of the object contributed in adjusting the grip force pattern to different phase delays of the load force patterns (note that the temporal improvement was done at a very fine scale). It would be difficult to assume that the object feedback was simply used as a sign to distinguish the two load force conditions (small and large delay conditions) since the same improvement was not observed in the both condition. On the other hand, one might suspect from the lack of improvement in the both condition (Fig. 5a: difference between both and control conditions) that the object feedback may have been processed as hand feedback in the object condition where the hand was invisible, and that this was critical in causing the improvement. Our second experiment, however, suggests that the lack of improvement in the both condition in the first experiment may be explained by a lack of attention to the object. Participants in the first experiment was instructed to move the cube (hand) with specified amplitude and movement frequency. Accordingly, it was likely that the participants directed their attention to the hand cursor rather than the object cursor when both cursors were shown. By instructing the participants to direct their attention to the object, the improvement was obtained even when the hand was also visible (Fig. 5b: difference between the attend-to-object and control conditions in experiment 2). Significant difference of grip force precedence between attend-to-hand and attend-to-object conditions in experiment 2 (also shown in Fig. 5b) suggested the involvement of visual attention. The pattern of hand movement suggested that the hand feedback was properly used by the motor system when provided even when the attention was directed to the object feedback. The amplitude of the hand trajectory increased in conditions without the hand feedback, possibly reflecting a larger safety margin for passing the edge markers or an intrinsic bias in hand localization when the hand is invisible. The amplitude was always smaller when the hand was visible (comparison between the both conditions and the control condition: *t*_15_ > 2.8, *p* < 0.02, *d* > 0.7), and did not differ by visual attention (no significant difference between the both conditions: *t*_15_ < 0.53, *p* > 0.6, *d* < 0.14).

Finally, the results of experiment 2 were replicated with a different set of participants while their fixations were rigorously monitored with an eye tracker (see section S2 in the Supplementary Information for details). This further confirmed our theory that visual attention, rather than differences in eye movements or visual inputs, caused the improvement in grip force timing.

### Force estimation based on multimodal cues

While our study focused on the contribution of visual feedback, we can assume several other information sources for the force estimation. First, earlier studies^1,4^ have suggested the contribution of somatosensory feedback. The observation in the first experiment that the participants were able to compensate for the trial-by-trial changes in load force timing without visual feedback may represent the contribution of somatosensory feedback. Secondly, Körding *et al*.^17^ showed that magnitude estimation of perturbation forces applied to a moving hand depends on Bayesian prior which reflects the statistical distribution of the experienced force magnitudes. This suggests the involvement of the prior.

Considering these findings, one may speculate that the temporal pattern of the experienced load force is estimated by integrating a Bayesian prior, a somatosensory estimate, and a visual estimate of the pattern. In line with this view, pattern of grip force precedence, measured in our two experiments, can be explained by assuming a linear summation of presumed prior and sensory estimates (see section S3 of the Supplementary Information for details). For instance, the general precedence of grip force relative to the load force, found in all the observed conditions, can be explained by assuming a Bayesian prior which corresponds to the load force pattern of a hand-held rigid object (note that the load force pattern is delayed in our case). If we assume that the inertia of the object is estimated from the object cursor when visual attention is directed to it, this would provide a visual estimate of the actual load force timing. Integration of this estimate would explain the improvement in grip timing under the object condition in experiment 1 and the attend-to-object condition in experiment 2. While several earlier studies^18,19^ did not find significant effect of visual attention to sensory integration, these studies observed estimation processes for spatial attributes whereas our study did not. Whether attention influences sensory integration varies depending on the observed domain and context^20^.

### Inverse dynamics computation for load force prediction

So far we have shown that visual feedback of object motion improves the load force estimation that underlies the grip force generation. This was consistent with the hypothesis that we can estimate the dynamic forces of objects from their visual motions. The question remains on how this estimation process was acquired and performed. Studies on force-field adaptation showed that providing different visual feedback of controlled object^21^ as well as attention to different control points of the object^22^ facilitates acquisition of separate internal models of force patterns. Visual feedback of object may have been associated with corresponding inertial load force patterns. Considering the Newton’s laws of motion, an effective strategy for encoding the association is to learn the correlation between the object acceleration and the load force. Then, this learned correlation can be used to estimate the load force pattern of various load motions.

Several recent studies seem to be consistent with this hypothesis. First, our earlier study showed that resistive sensation experienced while moving a delayed cursor correlates with amount of exposure to the forward acceleration of the cursor^6^. Secondly, while earlier behavioral studies^23–25^ have assumed, based on observations that slower velocity modulations are more easily detected, that the visual system detects accelerations only by cognitive processes which compares velocities over time, a recent study^26^ suggested existence of a hard-wired acceleration detector based on observation that we are highly sensitive to rapid accelerations when attentional tracking is prevented. Finally, neural recording in the medial superior temporal (MST) area during ocular following response have suggested that information of retinal slip acceleration is encoded in this area^27^.

Interestingly, a recent study^26^ has also shown that visual attention to the target facilitates the detection of velocity modulation when its temporal frequency is around 1 Hz. This could explain the effect of visual attention to the object cursor since the Bayesian framework predicts that improving the accuracy of visual estimate would decrease the grip precedence.

### Significance of the visual cue in force estimation

Although earlier studies demonstrated significant impact of visual feedback in our force estimation, one may still be suspicious about the need of the visual cue. Theoretically, the visual feedback can have an advantage over the somatosensory feedback. Many studies on human force/weight perception have shown the difficulty of force estimation from somatosensory signals. For instance, force estimation from the cutaneous signals is subject to changes in the state of contact because of different skin deformation. The significance of this issue is demonstrated in the fact that weight of a pinched object is perceived differently depending on the state of contact such as texture^28^ and shape^29^. Similarly, estimation of load forces from proprioceptive signals is subject to changes in arm posture as well as involved muscles. Earlier studies have revealed anisotropy in perceived forces^30^ as well as difficulty of matching the intensity of forces when different muscles are involved^31^. Even if the somatosensory feedback would have high sensitivity to the applied forces, calibration is needed to obtain an extrinsic representation of force, essential for dexterous motor control. The visual cue of force could play a major role in obtaining such representation^6^.

### Summary

To summarize, our study revealed that seeing and attending to an object that one moves can contribute to adjusting our grip force to its inertial load force. The pattern of changes in grip force pattern was consistent with a hypothesis that visual motion of the object provides an estimate of its inertial reaction force and that the visual estimate is integrated with the somatosensory estimate and a Bayesian prior. Our study provides evidence to suggest that grip-load force coupling does not simply reflect synergetic coupling between hand and arm muscles but rather results from multimodal estimation of the load timing. It also provides an objective evidence to suggest that the human brain is capable of inverse dynamics computation, the estimation of force from motion. As for future plan, we wish to observe in further depth the Bayesian estimation process in grip force control.

## Methods

### Participants

Twelve participants (2 males and 10 females; mean age = 29.2 years old) attended experiment 1 and sixteen (9 males and 7 females; mean age = 27.2 years old) attended experiment 2 after giving informed consent. Different set of participants contributed to the two experiments (no overlap). All participants were right-handed, had no reported neurological disorder, and naïve to the purpose of the study. Recruitment of participants and experimental procedures were approved by the NTT Communication Science Laboratory Research Ethics Committee and were conducted in accordance with the Declaration of Helsinki.

### General task setting

Figure 2 illustrates the general design of our experiment. As shown in Fig. 2a, participants sat in front of a table and pinched a cube attached to an end of a haptic device (Phantom premium 1.5 A, Geomagic Inc.) placed below the table. By moving the cube, participants moved a virtual object (mass) attached to the cube with a damped spring (i.e., spring-mass-damper system, Fig. 2b). The position of the object and its inertial reaction force was calculated at 1 kHz using the 4^th^-order Runge-Kutta method, and the force was applied to the cube. Two 3 degree-of-freedom force sensors (USL06-H5-50N, Tec Gihan Co. Ltd.) were attached to the front and back side of the cube to measure the load and grip forces. Measured load force delayed from the simulated load force by 44.3 ± 10.3 and 22.0 ± 10.2 ms in the small and large delay conditions (mean ± s.d. averaged across all visual conditions and participants), possibly due to the property of the haptic device. The average of these delays was approximately equal to the delay of the visual feedback, and the temporal discrepancy between the load force and the visual feedback was estimated to be below 5 degrees of phase difference in both load force conditions. Smooth sandpapers (#5000 Silicon Carbide 991 A SOFTFLEX) were attached to the contact surfaces. A linear slider constrained the trajectory and posture of the cube so that trajectory was restricted to a straight line and measured grip force would always be perpendicular to the hand trajectory and the load force.

Hand (cube) and object positions were displayed at 60 Hz as red and blue dots (radius: 6.5 mm) on a LCD display (RDT261WH, Mitsubishi Electric Co.) placed horizontally on the table. The delay of the display, measured at the beginning of each trial using a set of photodiodes, was 33.7 ± 3.1 ms (mean ± s.d. averaged across all conditions and participants). Screen image was adjusted so that displayed position of the hand and object coincide with the actual position of the cube and the computed position of the object. A fixation point was shown at the center of the screen 33 mm above the specified trajectory of the hand and object. Participants were instructed to fixate on the fixation point from a distance of approximately 300 mm in both experiments. The fixation was visually monitored throughout the experiments. In some occasions, participant made a smooth pursuit to follow the cursor that they controlled or made a saccade to either the cursor or the edge markers. In such cases, we warned the participant not to make any eye movements and rerun the experiment block. In experiment 2, attention condition was specified by the color of the fixation point and there were few cases where we had to warn the participants. Markers at the left and right edges (75 mm from the center) of the trajectory changed its color from light gray to dark gray (no change in contrast relative to background) when the distance of the cube from the center exceeded the distance of the edge markers from the center. This enabled the participants to adjust their movement amplitudes even when neither of the visual feedback sources were provided. It is important to note that this provided only binary information on hand location (whether the hand was inside or outside the area). Accordingly, if we assume a force estimation process based on prediction error of hand position (Fig. 1b), the markers would not provide information on the temporal pattern of the load force.

The task was to move the cube back-and-forth between the two markers with slight overshoots at a fixed movement frequency of 1 Hz specified by periodic beeps. Participants were told to move the cube along the linear slider. All participants were engaged in a training block before the main experiment to become capable of moving the cube with the specified trajectory.

Mechanical properties of the simulated system were selected so that the object follows the hand with a constant phase delay when the hand moved at the specified frequency (upper panel of Fig. 2c). Assuming hand position *x* [m], object position *y* [m], mass of the virtual object *m* [Kg], damping factor of spring *d* [Ns/m], and stiffness of spring *k* [N/m], as indicated in Fig. 2b, the following equation can be obtained using the Newton’s laws of motion:

$${\rm{m}}\ddot{{y}}=k(x-y)+d(\dot{x}-\ddot{y}).$$

Accordingly, the transfer function of the system *G*(*s*) is:

$${\rm{G}}({\rm{s}})=\frac{Y}{X}=\frac{ds+k}{m{s}^{2}+ds+k}.$$

When the hand follows a sinusoidal trajectory with a constant amplitude *A* [m], movement frequency ω [rad/s],

$$x(t)=\mathrm{Acos}{\rm{\omega }}{\rm{t}},$$

The trajectory of the object would be

$$y(t)=|{\rm{G}}({\rm{j}}{\rm{\omega }})|{\rm{Acos}}(\omega t+\angle G(jw))$$

where

$$\{\begin{array}{rcl}|G(j\omega )| & = & \frac{\sqrt{{k}^{2}+{d}^{2}{\omega }^{2}}}{\sqrt{{(k-m{\omega }^{2})}^{2}+{d}^{2}{\omega }^{2}}}\\ \angle G(j\omega ) & = & arctan(\frac{d\omega }{k})-arctan(\frac{d\omega }{k-m{\omega }^{2}})\end{array}$$

By setting ${\rm{k}}=\frac{m{\omega }^{2}}{2}$ and ${\rm{d}}=\frac{m\omega }{2}\,\tan (\frac{\pi -\theta }{2})$, the object trajectory was controlled to follow the hand position with a phase delay of θ [rad] such that

$${\rm{y}}(t)=Acos(\omega t-\theta ).$$

Two load force conditions with different delays (small and large delay conditions corresponding to θ of 80 and 100 degrees respectively) were tested for each visual feedback condition.

Each trial of the experiments consisted of 10 s of the specified movement. The trial started when the hand turned at the right edge. After each trial, visual feedback was removed for a short period (longer than 200 ms) until the next trial began. Participants continued the cyclic movement within each block which consisted of multiple trials but always ended within 10 minutes. Resting period was provided in between each block.

### Experiment 1: Contribution of hand and object visual feedback

In the first experiment, we examined how visual feedback of hand and object positions each contribute to the coordination of the grip force. Here, we examined four visual feedback conditions: a condition with only the hand visual feedback (hand condition), a condition with only the object visual feedback (object condition), a condition with both hand and object visual feedback (both condition), and a condition without the two visual feedback sources (control condition). In all conditions, participants were instructed to control the cube with the specified movement amplitude and frequency. In the object condition, they were required to simultaneously control the object so that it would also move with the specified amplitude and frequency. Together with the load force condition, there were 8 conditions in total. The conditions were experienced in pseudo-random order within each block. Each block contained 4 trials for each condition and there were 8 blocks (32 trials for each condition in total). The experiment lasted for approximately 2 hours.

### Experiment 2: Relevance of attention to hand and object visual feedback

In the second experiment, we examined how attending to either the hand cursor or the object cursor influenced the coordination of the grip force. Here, we examined three visual feedback conditions: a condition in which both the hand and object positions are shown and the participants were instructed to control the hand to move with the specified amplitude and frequency (attend-to-hand condition), a similar condition in which the participants were instructed to control the object to move with the specified amplitude and frequency (attend-to-object condition), and the condition without the two visual feedback sources (control condition). In the attend-to-object condition, the color of the edge markers changed according to the position of the object rather than the cube. Together with the load force condition, there were 6 conditions in total. The conditions were experienced in pseudo-random order within each block. Each block contained 4 trials for each condition and there were 8 blocks (32 trials for each condition in total). The experiment lasted for approximately 1.5 hours.

### Data analysis

The positions of the hand (cube) and the virtual object as well as the load and grip forces were recorded at 1 kHz and low-pass-filtered at 7 Hz using a fourth-order, no-lag, Butterworth filter. Then, the following values were calculated for each trial and averaged across all trials within each condition. In order to examine how the visual feedback condition changed the hand trajectory, width and cycle of the hand trajectory were calculated as average peak distance of the cube from the center and the average interval of the cube crossing the center, respectively. In order to calculate the timing of the hand trajectory relative to the beeps, we first assumed a sinusoidal hand trajectory which reaches its ends in synchrony with the beeps. The timing was then calculated as the temporal displacement which maximizes the cross-correlation between the actual and the assumed hand trajectories. To assess whether the object behaved as intended, gain and delay of the object motion relative to the cube motion were calculated. The gain was calculated as ratio between average cube distance and average object distance from the center, whereas the delay was calculated as a temporal displacement which maximizes the cross-correlation between the two trajectories. Finally, in order to examine how each feedback contributed to synchronizing the grip force to the load force, we calculated the temporal discrepancy between the two forces. This was calculated as a temporal displacement which maximizes the cross-correlation between the two forces^32^. All cross-correlations were calculated for the temporal window of 9 s starting from the first turn at the left edge after trial onset. After obtaining the above-mentioned values for each participant and condition, two-factor repeated-measures ANOVA was applied to examine the effects of the visual (4 levels for experiment 1 and 3 levels for experiment 2) and load force conditions (2 levels for both experiments). Ryan’s method^14^ was for multiple comparison.

## Supplementary information

Supplementary Information

## Data Availability

The datasets generated and/or analyzed during the current study are available from the corresponding author on reasonable request.
