# Supplementary material for: Better grip force control by attending to the controlled object: Evidence for direct force estimation from visual motion
Source: Sci Rep. 2019 Sep 11;9:13114. doi: 10.1038/s41598-019-49359-8 (PMC6739397; doi:10.1038/s41598-019-49359-8)
Supplement: Supplementary file 1 — Supplementary Information [file 41598_2019_49359_MOESM1_ESM.pdf]

Better grip force control by attending to the controlled object: Evidence for direct force estimation from visual motion

Shinya Takamuku[1,*] and Hiroaki Gomi[1]

[1]NTT Communication Science Laboratories, 3-1 Morinosato Wakamiya, Atsugi, Kanagawa, 243-0198, Japan

*Corresponding author. Dr. Shinya Takamuku, NTT Communication Science Laboratories, 3-1 Morinosato Wakamiya, Atsugi, Kanagawa, 243-0198, Japan
takamuku.shinya@lab.ntt.co.jp; shinya.takamuku@gmail.com

Contents:

S1. Two hypotheses that explain the temporal shift of grip force pattern observed in the study by Sarlegna et al.[1]

S2. Replication of attention effect under monitored fixation

S3. Bayesian estimation theory for explaining the grip-load force coupling

S1. Two hypotheses that explain the temporal shift of grip force pattern observed in the study by Sarlegna et al. [1]

S1.1 Summary

Sarlegna et al.[1] reported changes in grip force pattern caused by a delay in visual feedback as evidence for visual contribution to grip force adjustments. Here we show that two potential visual contributions to load force estimation can both account for this finding; one based on visual estimation of inertia from object motion[1,2] and the other based on perturbation force estimation based on prediction error of hand position, the latter process often assumed in contexts of motor learning[3,4] and human-computer interaction[5,6]. Importantly, the former contribution is based on *inverse* dynamics computation whereas the latter is based on *forward* dynamics computation. Therefore, the distinction relates to the fundamental question on whether the human brain also solves the computational problem of inverse dynamics[2]; the estimation of force from motion. In the following sections, we will first describe the finding reported by Sarlegna et al. (S1.2) and then describe the two possible hypotheses (S1.3 and S1.4). Finally we will describe the critical distinction between the two hypotheses which we have addressed in our study (S1.5).

S1.2 Finding

Top and bottom panels in Fig. S1 illustrate the setup used in the study reported by Sarlegna et al.[1]. Participants pinched a force sensor which was attached to a wall with an elastic cord. They repeatedly stretched the cord while observing a cursor on a computer monitor that indicated the position of the pinched sensor. When the motion of the cursor was delayed relative to the motion of the sensor, grip force applied to the sensor tended to precede the load force. The temporal precedence increased as the delay increased within the observed range (phase delay of $0 < \theta \le \pi/3$ [rad]).

S1.3 Cursor-inertia hypothesis [1]

In order to explain the observed temporal shift of grip force pattern, Sarlegna et al. hypothesized that the participants interpreted the delayed cursor as indicating a mass attached to the sensor with a damped spring (upper middle panel of Fig. S1), and adjusted their grip force to compensate for the imaginary inertial force of the virtual mass.

Let's assume that the position of the sensor, x [m], at time t [s] follows a sinusoidal trajectory with a movement frequency of ω [rad/s]

$$x(t) = A\sin(\omega t).$$

The actual load force applied to the grip (F_p [N]) at time t can then be calculated as

$$F_p(t) = -k_c(x - x_0) = -Ak_c\sin(\omega t) + k_c x_0$$

where k_c [N/m] and x_0 [m] represent the stiffness and the equilibrium point of the cord.

The position indicated by the cursor, y [m], which lags behind the actual sensor position by a phase delay of θ [rad], would be

$$y = A\sin(\omega t - \theta).$$

If the inertia of the virtual mass is assumed to be m [kg], the inertial force of the mass applied to the sensor (F_{vi} [N]) at time t can be calculated from its acceleration $\ddot{y}$ [m/s^2] based on the Newton's laws of motion as follows:

$$F_{vi}(t) = -m\ddot{y} = Am\omega^2\sin(\omega t - \theta).$$

Accordingly, the combined load force (F_{ci} [N]) at time t would be

$$F_{ci} = F_p + F_{vi} = A_{ci}\sin(\omega t + \theta_{ci}) + const.,$$

where

$$A_{ci} = A\sqrt{k_c{}^2 + m^2\omega^4 - 2mk_c\omega^2\cos\theta}$$

$$\theta_{ci} = \tan^{-1}\left(\frac{-m\omega^2\sin\theta}{m\omega^2\cos\theta - k_c}\right) + \begin{cases} 0 & if \quad m\omega^2 - k_c \geq 0 \\ \pi & if \quad m\omega^2 - k_c < 0 \end{cases}$$

If we assume that the virtual inertia is significantly smaller compared to the stiffness of the elastic cord such that $m < k_c/\omega^2$, the phase shift from the phase of the actual load force pattern (π) would be

$$\theta_{ci} - \pi = \tan^{-1}\left(\frac{m\omega^2\sin\theta}{k_c - m\omega^2\cos\theta}\right) \quad .$$

As long as the cursor lags behind the hand position (i.e., $0 < \theta \leq \pi$), both the numerator and the denominator are positive, so the phase shift would be positive. Furthermore, it will monotonically increase within the range observed in the study ($0 < \theta \leq \pi/3$). Assuming that the grip force is adjusted based on the predicted load force, the model explains the temporal precedence of the grip force relative to the actual load force which scales with the delay.

S1.4 Perturbation model

While the theory mentioned above explains the temporal precedence of the grip force from the load force, we noticed that a simple assumption, often made in models of force-field adaptation[3,4], also explains the phenomenon. This assumes that participants estimate the strength of the force applied to their hands from the error of cursor position caused by the applied force (illustrated in the lower middle panel of Fig. S1; see Introduction in the main text for further detail). In the situation observed in the study by Sarlegna et al., perturbation force (F_{vp} [N]) at time *t* can be estimated from the error of cursor position caused by the delay (*y* - *x*) as

$$F_{vp}(t) = k_a(y - x) = Ak_a\{\sin(\omega t - \theta) - \sin(\omega t)\}$$

where k_a [N/m] represents the stiffness of the arm at its endpoint (hand). In this case, the combined load force (F_{cp} [N]) at time t would be

$$F_{cp} = F_p + F_{vp} = A_{cp}\sin(\omega t + \theta_{cp}),$$

where

$$A_{cp} = A\sqrt{(k_a + k_c)^2 + k_a{}^2 - 2(k_a + k_c)k_a\cos\theta}$$
$$\theta_{cp} = \tan^{-1}\left(\frac{k_a\sin\theta}{(k_a + k_c) - k_a\cos\theta}\right) + \begin{cases} 0 & if \quad k_a\cos\theta - (k_a + k_c) \geq 0 \\ \pi & if \quad k_a\cos\theta - (k_a + k_c) < 0 \end{cases}.$$

Considering that k_a and k_c are both positive, $k_a\cos\theta - (k_a + k_c)$ should always be negative. Therefore, the phase shift from the phase of the actual load force (π) would be

$$\theta_{cp} - \pi = \tan^{-1}\left(\frac{k_a\sin\theta}{(k_a + k_c) - k_a\cos\theta}\right).$$

Since both the numerator and the denominator are positive, the phase shift would always be positive. Furthermore, if we assume that the virtual stiffness of the arm is significantly smaller than the stiffness of the cord, it will monotonically increase at the observed range of $0 < \theta \leq \pi/3$. Therefore, the model also explains the temporal precedence of the grip force.

S1.5 Relation between the two hypotheses

The force estimation process assumed by Sarlegna et al. would require an *inverse dynamics model* to estimate the inertial force from the motion of the delayed cursor. Meanwhile, the force estimation process described in section

S1.3 would require a *forward dynamics model* to predict the cursor position from which the error would be calculated. The critical difference between the two processes is whether the cursor is interpreted as an object motion or as a hand motion. Our study reported in the main text suggests that the cursor is interpreted as object motion in the context of force estimation and therefore supports the former hypothesis.

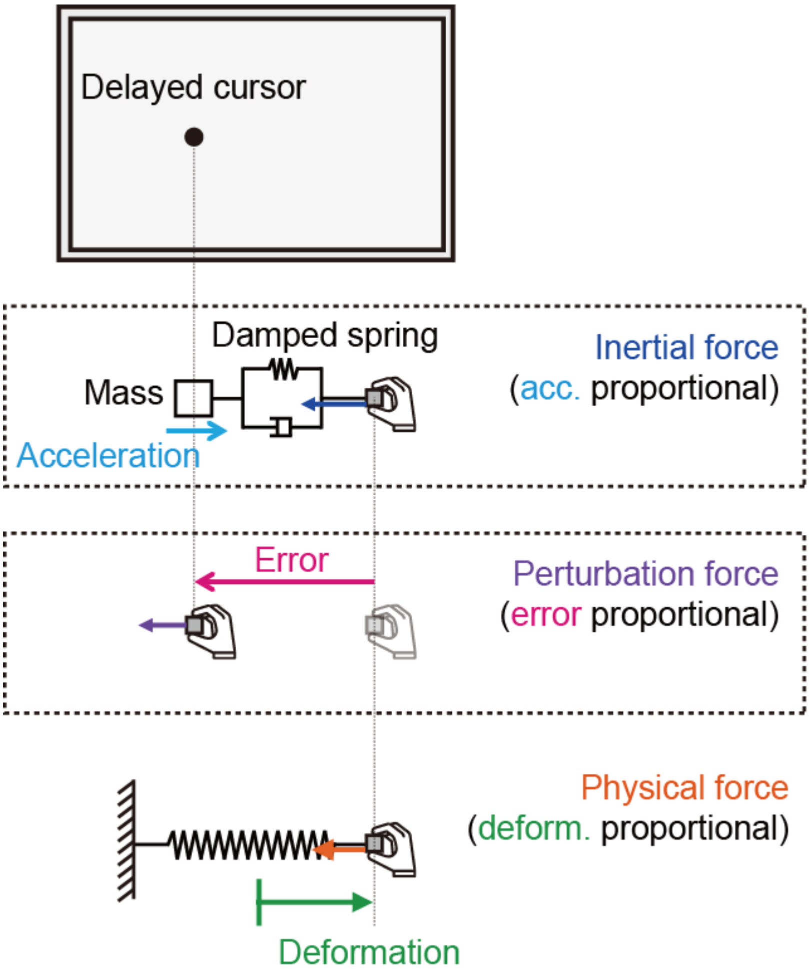

Fig. S1 Hypotheses for the result reported by Sarlegna et al.

S2. Replication of attention effect with monitored fixation

S2.1 Summary

Considering the earlier finding[7] that visual attention and saccadic eye movements are not completely independent, one could claim that participants may have made saccades to the cursors when asked to direct their attention to them in experiment 2. While the fixations of the participants were visually monitored by the experimenter throughout the experiment, it was difficult to detect small deviations from the fixation or instantaneous saccades to the cursor. To rule out the possibility that any difference in eye movements or visual input caused the improvement in grip force timing, we replicated the results of experiment 2 using a different set of participants while their fixations were monitored using an eye tracker (SR Research EyeLink II).

S2.2 Method

Ten participants (1 male and 9 female; mean age = 36.5 years old) contributed to the experiment. The task given to the participants were identical to that in experiment 2. They placed their chin on a chin rest. An eye tracker was attached to their head and their left eye was monitored throughout the experiment. Based on a 5-point calibration in the beginning of every experiment block, gazed position on the screen was calculated and recorded at 500Hz. In between each trial, the fixation flickered to force the participant to fixate on the fixation point. The gaze positions during these intervals were used to remove the drifting component. After removing the blinks, the trajectory of gaze position was checked for each trial, and trials in which the gaze position exited from the predefined fixation area (area with distance from the fixation smaller than 50 pixels) were excluded from further analysis. The fixation was located at the center of the screen (x = 0, y = 0 pixels). The cursor trajectory was 100 [pixels] below the fixation (y = -100 pixels). The side markers were 175 [pixels] apart from the fixation (x = -175, 175 pixels).

S2.3 Result

Fig. S2a shows the normalized heat map of gaze position for each condition of a representative participant. The fixation distributed around the fixation cross for all the observed conditions. Two-factor repeated measures ANOVA (factors: visual and load conditions) revealed that neither the visual condition, the load

condition, nor the interaction between the two conditions had a statistically significant effect on the number of excluded trials ($p > 0.4$ for all effects), x and y gaze positions averaged across all trials ($p > 0.1$ for all effects), or the standard deviation of x and y gaze positions calculated for each trial and averaged across all trials ($p > 0.2$ for all effects).

Fig. S2b shows the temporal precedence of grip force relative to the load force averaged across all participants. Here, the ANOVA revealed significant effects of visual condition ($F_{2,18} = 8.93,\ p = 2.0 \times 10^{-3},\ \eta_p^2 = 0.50$), load force condition ($F_{1,9} = 29.4,\ p = 4.0 \times 10^{-4},\ \eta_p^2 = 0.77$), and the interaction between the two conditions ($F_{2,18} = 4.62,\ p = 2.4 \times 10^{-2},\ \eta_p^2 = 0.34$). Post-hoc analysis (Ryan's method) among the visual conditions showed that the temporal precedence was smaller than the control condition when attention was directed towards the object feedback ($t_9 = 4.0,\ p = 8.3 \times 10^{-4},\ d = 1.26$), but not when it was directed towards the hand feedback ($t_9 = 0.83,\ p = 0.42,\ d = 0.26$). The precedence differed significantly by visual attention (difference between both conditions: $t_9 = 3.17,\ p = 5.2 \times 10^{-3},\ d = 1.0$). Namely, the improvement in grip force timing was observed even under monitored fixation. This supported the idea that the improvement is associated with visual attention, rather than eye movements.

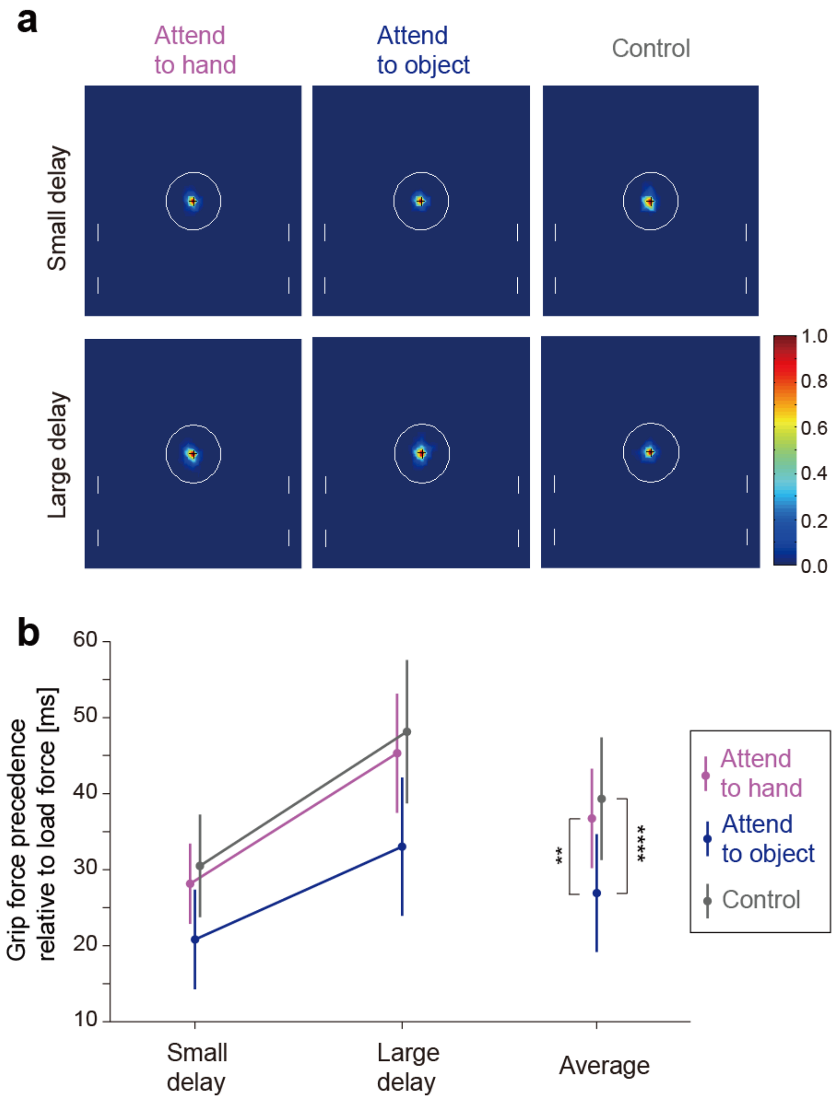

Figure S2 Replication of attention effect with monitored fixation. **a**: Normalized heat map of gaze position for each condition of a representative participant. The black cross, the white vertical lines, and the white circle represent the fixation, the side markers, and the fixation area, respectively. **b**: Temporal precedence of grip force relative to load force averaged across all participants. Colors indicate visual condition. Error bars denote standard errors across participants. Asterisks along the vertical bars indicate significant difference between the visual conditions. Multiple comparison was controlled based on the Ryan's method. ***, and **** denote $p < 0.005$, and $p < 0.001$, respectively.

S3. Bayesian estimation theory for explaining the grip-load force coupling

Körding et al.[8] showed that magnitude estimation of perturbation forces applied to a moving hand depends on Bayesian prior which reflects the statistical distribution of the experienced force magnitudes. The precedence of grip force relative to the load force, examined in our two experiments, can be explained by extending this theoretical framework to multimodal estimation of temporal patterns of load forces linked to self-motor action. Figure S3 illustrates the hypothesis. The task we consider for grip force control is to estimate the temporal pattern of load force associated with the hand action. First, we assume a Bayesian prior of the load force pattern. Considering that we often move a rigid object with a pinch grip, we assumed a Bayesian prior of the load force pattern which corresponds to that of a rigid object held in our hands. This predicts a load force synced with the hand acceleration. Integration of such a prior with a somatosensory estimate of the load force pattern, which should generally represent the actual pattern, predicts that the grip force would sync with the load force when we move a rigid object, but will precede the load force when moving an object with a lagged trajectory. This was actually what we observed. Earlier studies also reported that grip precedence is observed when moving a non-rigid object [9,10]. The framework also explains why the precedence was larger in the large delay condition (note that the difference from the prior is larger in this condition). We also found that grip precedence, averaged across all conditions, gradually decreased during the experiment (statistically significant correlation between trial number and grip precedence averaged across all conditions and participants in the 2nd experiment; $R = -0.5$,

$p = 3.3 \times 10^{-3}$). The decrease may represent a gradual update of the prior, although it may also reflect muscle fatigue. Secondly, we assume that the visual motion of the object provides an estimation of the load force pattern which is proportional to its acceleration[1,2] (inertial force of the cursor) when attention is directed to it. Since this is roughly equal to the actual load force pattern in our case, Bayesian integration of the visual estimate should decrease the precedence of the grip force. This explains the improvement in the object condition of experiment 1 and the attend-to-object condition in experiment2. Finally, the theory also predicts that delaying the cursor motion would increase the grip precedence when the actual load force acts in the opposite direction compared to the visually implied inertia of the cursor. This was the case in the study by Sarlegna et al.[6] and the increase in the grip precedence was actually observed.

While this theory remains to be tested, earlier studies have confirmed that our brain uses Bayesian integration to estimate the magnitude of forces applied to the reaching hand[8], grip force control relies on the statistical distribution of the experienced load forces[11] , and the Bayesian computation also applies to timing estimation based on visual cues[12].

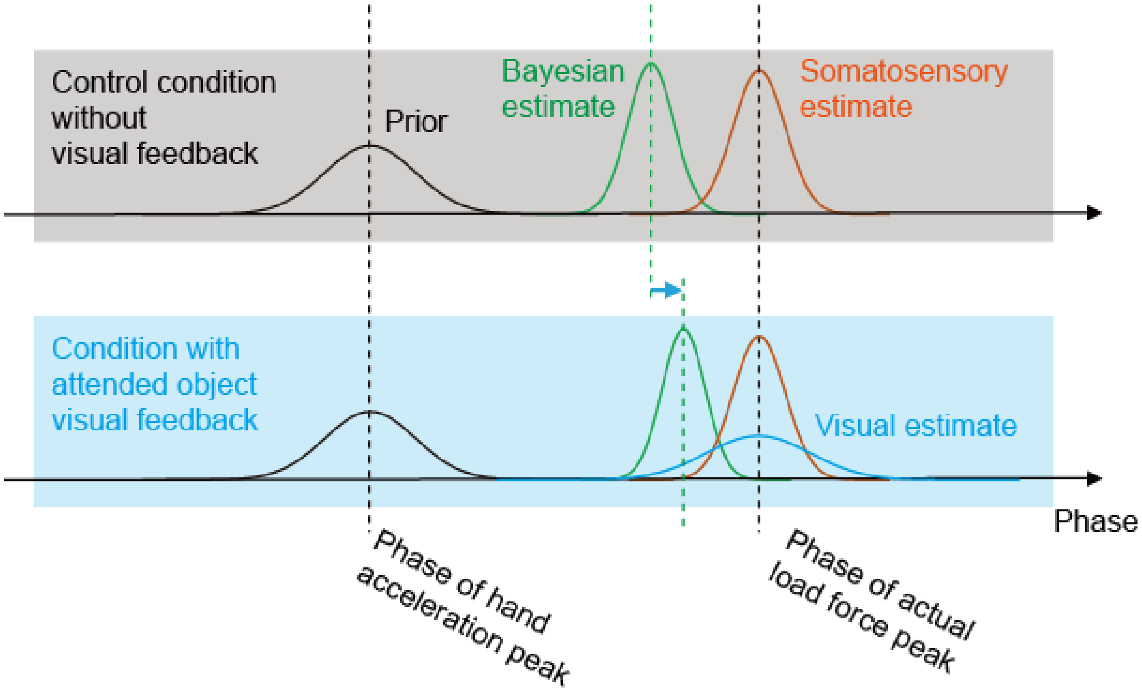

Figure S3 Bayesian account of grip force precedence. Top and bottom panels illustrate how the time of load force peak is estimated from multiple factors. The horizontal axis represents the phase lag of the load force peak relative to the hand acceleration. Distribution illustrated with black, orange, and blue lines illustrate the distributions of prior, somatosensory estimate, and visual estimate of the time of the load force peak. The distribution illustrated in green represents the Bayesian estimate of the load-force-peak time.

Reference

1. Sarlegna, F. R., Baud-Bovy, G. & Danion, F. Delayed visual feedback affects both manual tracking and grip force control when transporting a handheld object. *J. Neurophysiol.* **104**, 641–653 (2010).

2. Takamuku, S. & Gomi, H. What you feel is what you see: inverse dynamics estimation underlies the resistive sensation of a delayed cursor. *Proc. Biol. Sci.* **282**, (2015).

3. Scheidt, R. A., Dingwell, J. B. & Mussa-Ivaldi, F. A. Learning to move amid uncertainty. *J. Neurophysiol.* **86**, 971–985 (2001).

4. Marko, M. K., Haith, A. M., Harran, M. D. & Shadmehr, R. Sensitivity to prediction error in reach adaptation. *J. Neurophysiol.* **108**, 1752–1763 (2012).

5. Lécuyer, A., Burkhardt, J.-M. & Etienne, L. Feeling bumps and holes without a haptic interface: the perception of pseudo-haptic textures. in *Proceedings of the SIGCHI Conference on Human Factors in Computing Systems* 239–246 (ACM, 2004). doi:10.1145/985692.985723

6. Pusch, A., Martin, O. & Coquillart, S. HEMP-hand-displacement-based Pseudo-haptics: A Study of a Force Field Application and a Behavioural Analysis. *Int J Hum-Comput Stud* **67**, 256–268 (2009).

7. Deubel, H. & Schneider, W. X. Saccade target selection and object recognition: evidence for a common attentional mechanism. *Vision Res.* **36**, 1827–1837 (1996).

8. Körding, K. P., Ku, S. & Wolpert, D. M. Bayesian integration in force estimation. *J. Neurophysiol.* **92**, 3161–3165 (2004).

9. Danion, F., Descoins, M. & Bootsma, R. J. When the fingers need to act faster than the arm: coordination between grip force and load force during oscillation of a hand-held object. *Exp. Brain Res.* **193**, 85–94 (2009).

10. Flanagan, J. R. & Wing, A. M. The role of internal models in motion planning and control: evidence from grip force adjustments during movements of hand-held loads. *J. Neurosci.* **17**, 1519–1528 (1997).

11. Hadjiosif, A. M. & Smith, M. A. Flexible Control of Safety Margins for Action Based on Environmental Variability. *J. Neurosci. Off. J. Soc. Neurosci.* **35**, 9106–9121 (2015).

12. Miyazaki, M., Nozaki, D. & Nakajima, Y. Testing Bayesian models of human coincidence timing. *J. Neurophysiol.* **94**, 395–399 (2005).
